# Supplementary material for: Phase interface engineering enables state-of-the-art half-Heusler thermoelectrics
Source: Nat Commun. 2024 Jul 16;15:5978. doi: 10.1038/s41467-024-50371-4 (PMC11252142; doi:10.1038/s41467-024-50371-4)
Supplement: Supplementary file 1 — Supplementary Information [file 41467_2024_50371_MOESM1_ESM.pdf]

Supporting Information

Phase Interface Engineering Enables State-of-the-Art Half-Heusler Thermoelectrics

Author Information

Yihua Zhang[1,2,#], Guyang Peng[2,#], Shuankui Li[3,#], Haijun Wu[2,*], Kaidong Chen[1], Jiandong Wang[2], Zhihao Zhao[2], Tu Lyu[1], Yuan Yu[4], Chaohua Zhang[1], Yang Zhang[5,6], Chuansheng Ma[6], Shengwu Guo[2], Xiangdong Ding[2,*], Jun Sun[2], Fusheng Liu[1,*], and Lipeng Hu[1,*]

Affiliations

[1]College of Materials Science and Engineering, Shenzhen Key Laboratory of Special Functional Materials, Guangdong Research Center for Interfacial Engineering of Functional Materials, Guangdong Provincial Key Laboratory of Deep Earth Sciences and Geothermal Energy Exploitation and Utilization, Institute of Deep Earth Sciences and Green Energy, Shenzhen University, Shenzhen 518060, China

Yihua Zhang, Kaidong Chen, Tu Lyu, Chaohua Zhang, Fusheng Liu & Lipeng Hu.

[2]State Key Laboratory for Mechanical Behavior of Materials, Xi'an Jiaotong University, Xi'an 710049, China

Yihua Zhang, Guyang Peng, Haijun Wu, Jiandong Wang, Zhihao Zhao, Shengwu Guo, Xiangdong Ding & Jun Sun.

[3]School of Physics and Materials Science, Guangzhou University, Guangzhou 510006, China

Shuankui Li.

[4]Institute of Physics (IA), RWTH Aachen University, Sommerfeldstraße 14, 52074, Aachen, Germany

Yuan Yu.

[5]Electronic Materials Research Laboratory (Key Lab of Education Ministry) and School of Electronic Science and Engineering, Xi'an Jiaotong University, Xi'an 710049, China.

Yang Zhang.

[6]Instrumental Analysis Center of Xi’an Jiaotong University, Xi’an Jiaotong University, Xi’an 710049, China

Yang Zhang, Chuansheng Ma.

Contributions

[#]These authors contributed equally to this work.

Corresponding authors

Correspondence to: H. W. (email: wuhaijunnavy@xjtu.edu.cn) ORCID ID: 0000-0002-7303-379X

X. D. (email: dingxd@xjtu.edu.cn);

F. L. (email: fsliu@szu.edu.cn);

L. H. (email: hulipeng@szu.edu.cn);

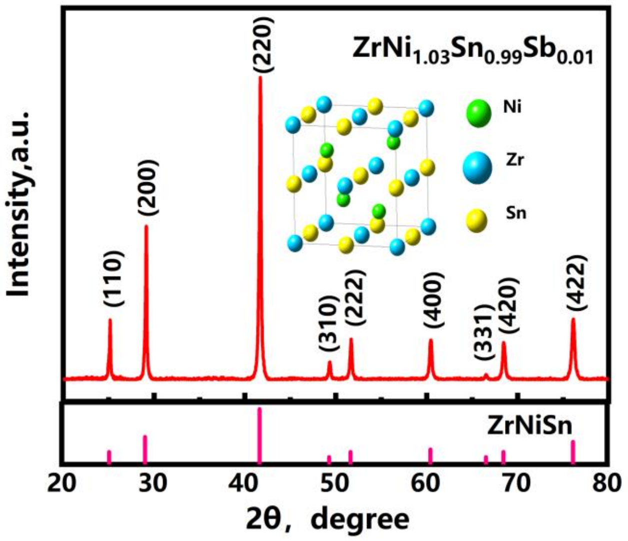

Fig. S1. XRD pattern of the as-prepared $ZrNi_{1.03}Sn_{0.99}Sb_{0.01}$ (ZNSS) powders.

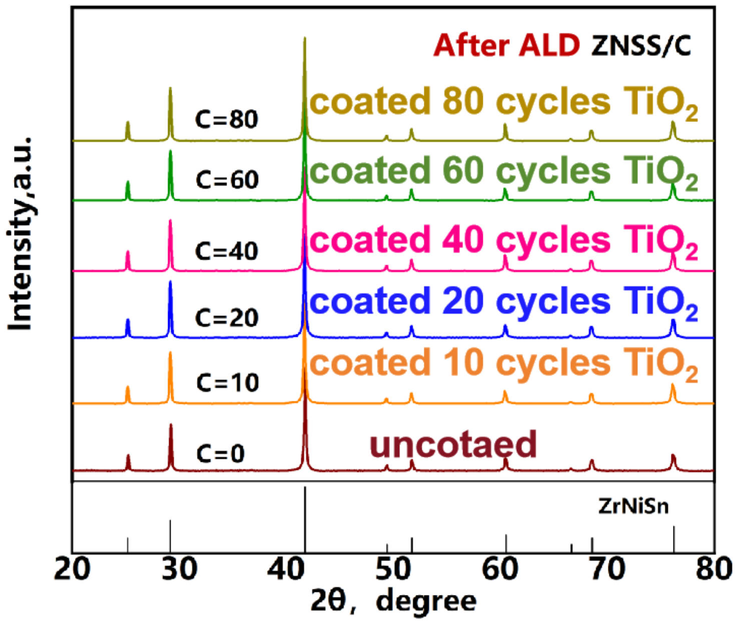

Fig. S2. XRD pattern of the samples with different ALD cycles before SPS process.

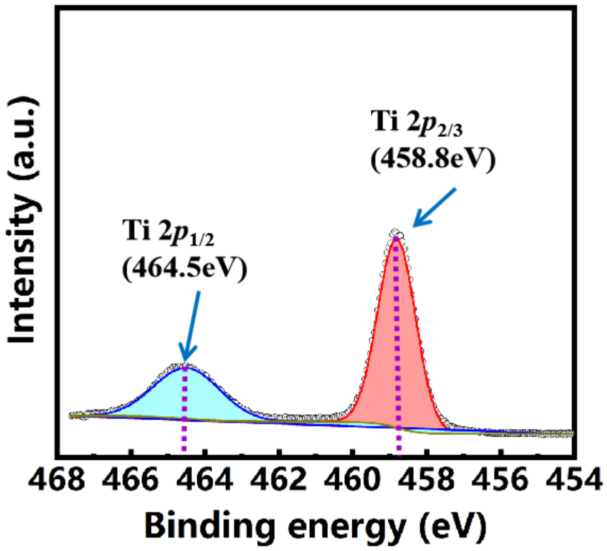

Fig. S3. XPS spectra of Ti 2$p_{1/2}$ and Ti 2$p_{1/3}$ core level for TiO_2 = 3.2 nm sample.[1]

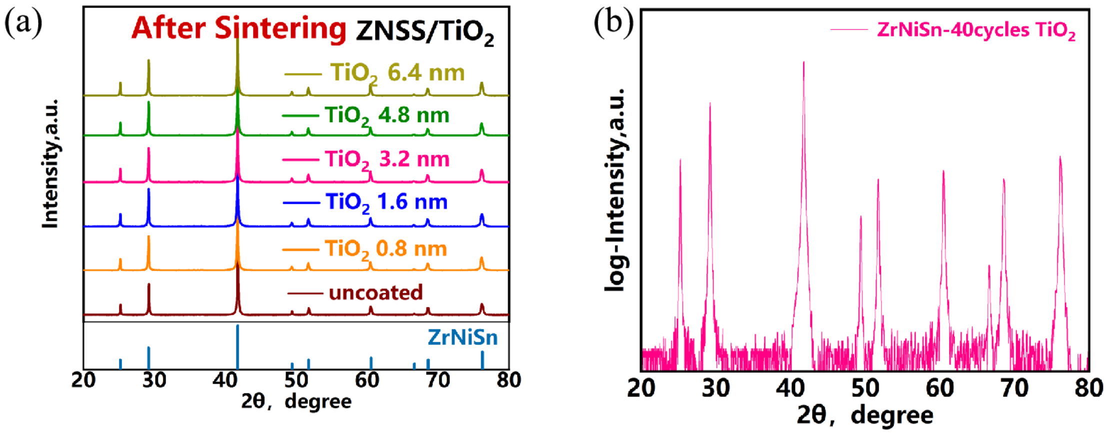

Fig. S4. (a) XRD pattern of the samples with different ALD cycles after SPS process; (b) XRD pattern (log mode) of TiO_2 = 3.2 nm sample after SPS process.

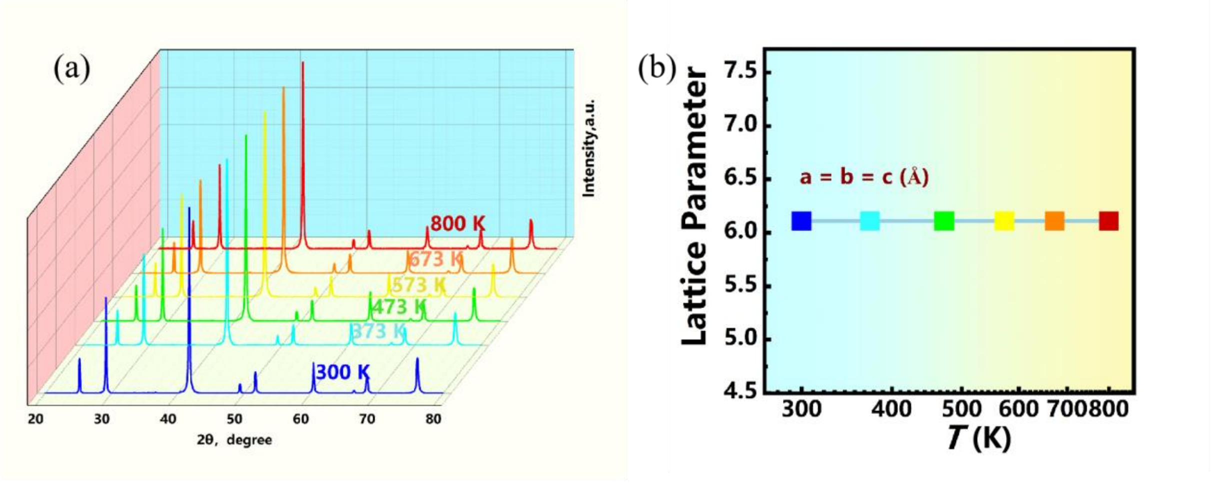

Fig. S5. (a) Variable temperature XRD patterns of TiO_2 = 3.2 nm samples and (b) the corresponding lattice parameters

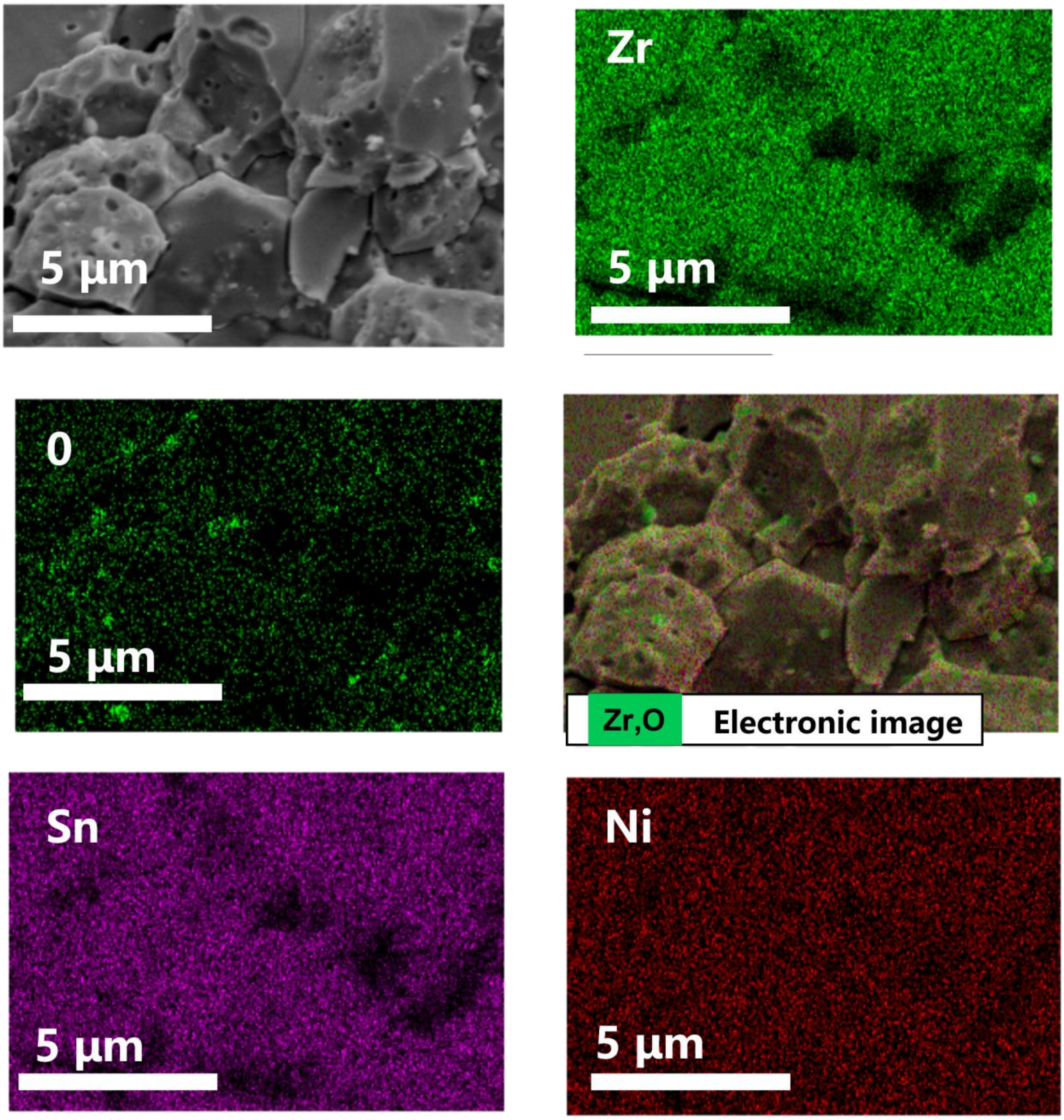

Fig. S6. Cross-sectional scanning morphology and EDS element distribution of TiO_2 = 3.2 nm sintered sample.

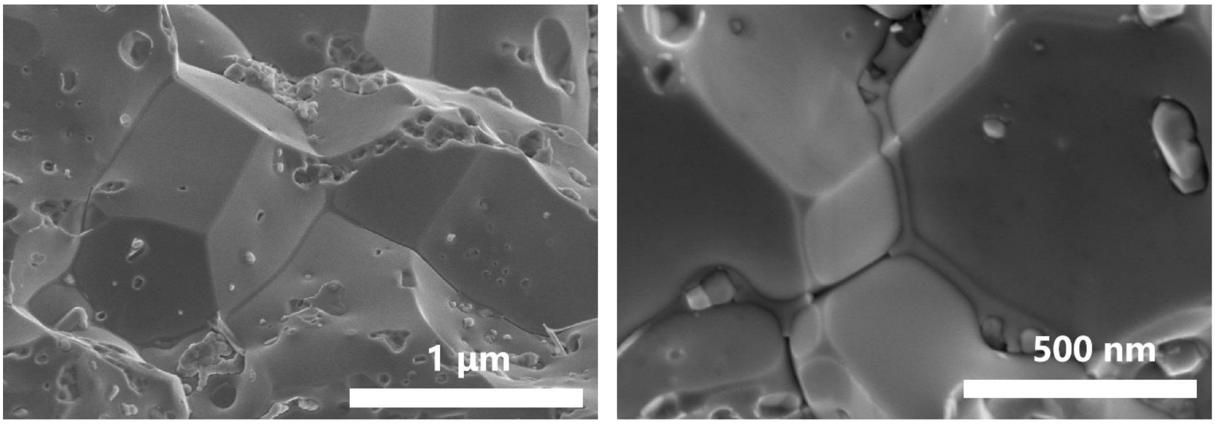

Fig. S7. Cross-sectional scanning morphology of TiO_2 = 3.2 nm sintered sample.

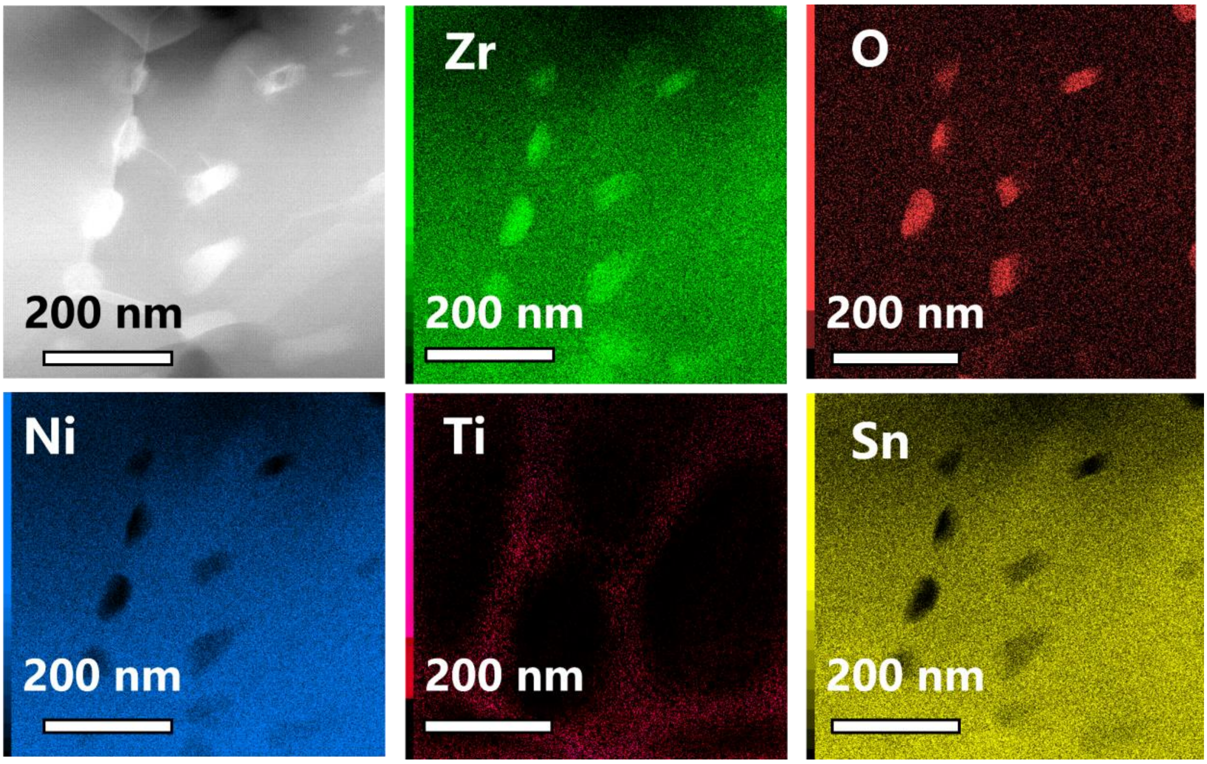

Fig. S8. ABF image and corresponding EDS mapping images of the ZrO_2 nanoparticle.

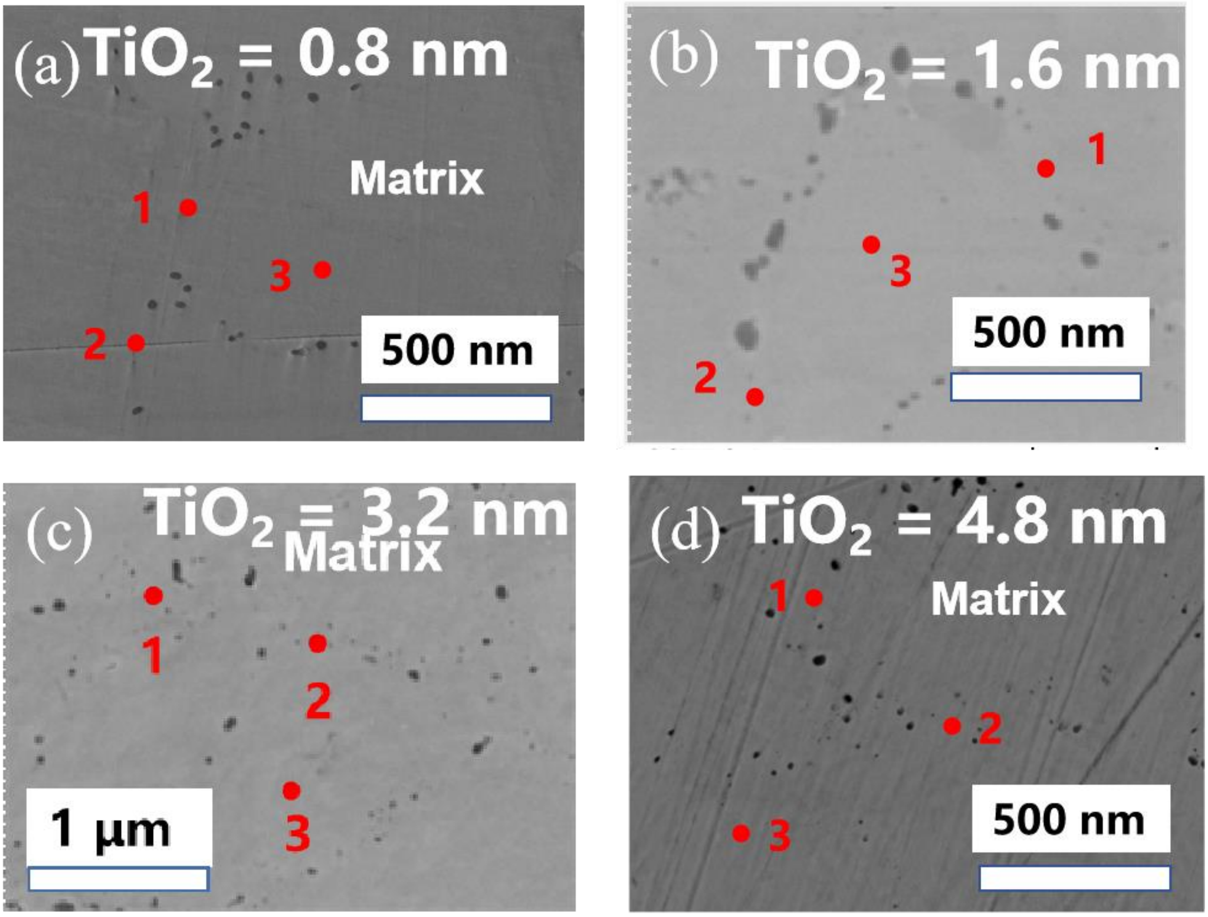

Fig. S9. EPMA backscattered electron image of TiO_2 = 0.8 nm, TiO_2 = 1.6 nm, TiO_2 = 3.2 nm, TiO_2 = 4.8 nm samples.

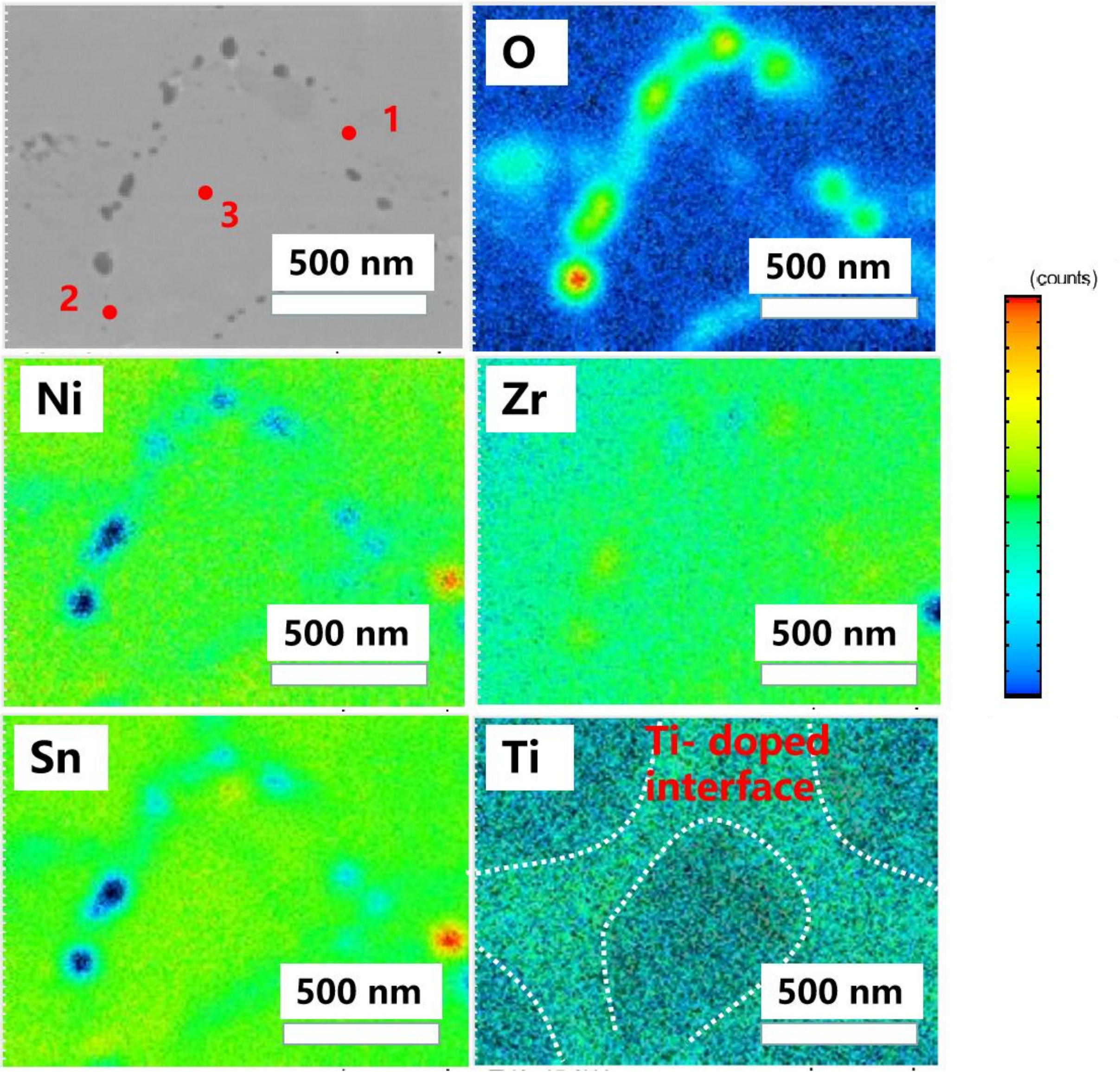

Fig. S10. EPMA backscattered electron image of TiO_2 = 1.6 nm sample and the corresponding EDS element of O, Zr, Ni, Sn, Ti maps (the color from blue to red indicates the content of elements from less to more).

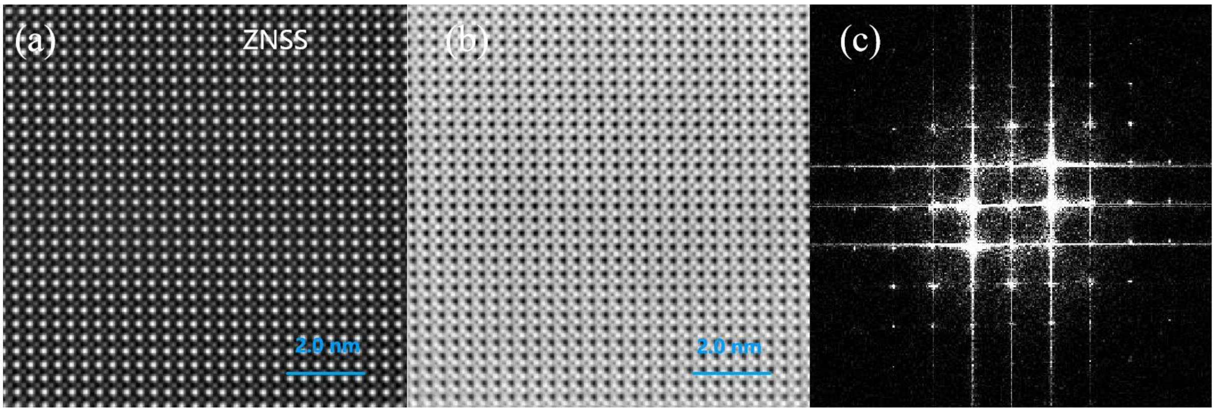

Fig. S11. (a) HADDF, (b) ABF and (c) FFT images of matrix ZNSS.

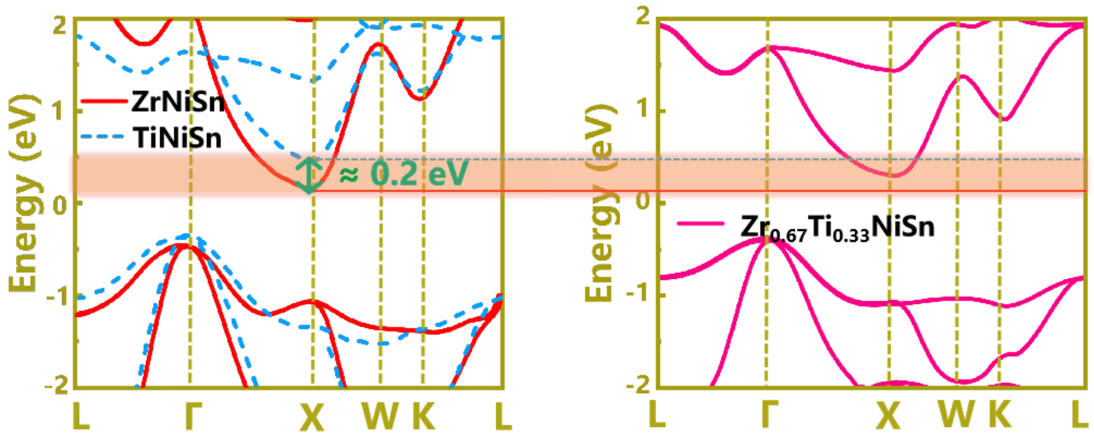

Fig. S12. Band structure diagram of (a) ZrNiSn, TiNiSn and (b) $Zr_{0.67}Ti_{0.33}NiSn$.

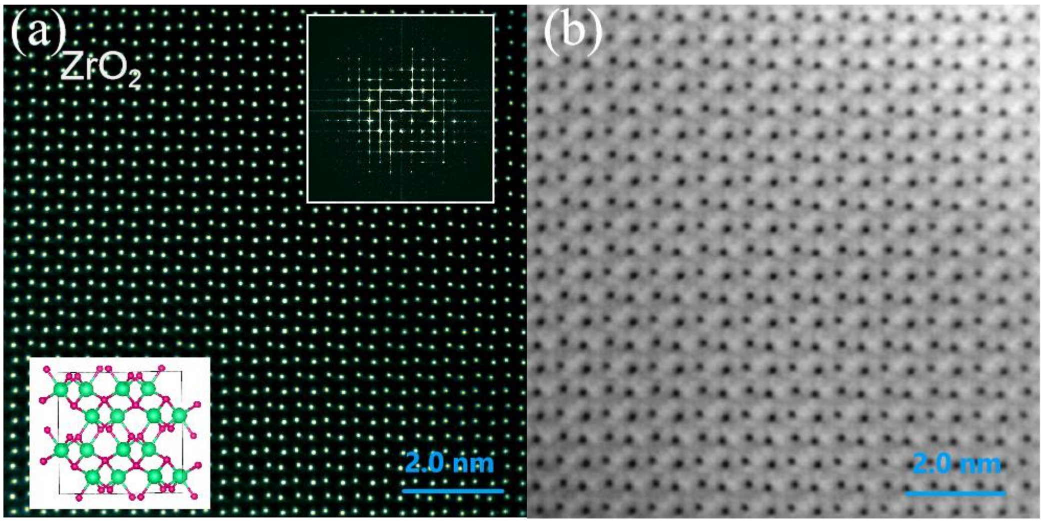

Fig. S13. (a) HADDF and (b) ABF images of ZrO_2.

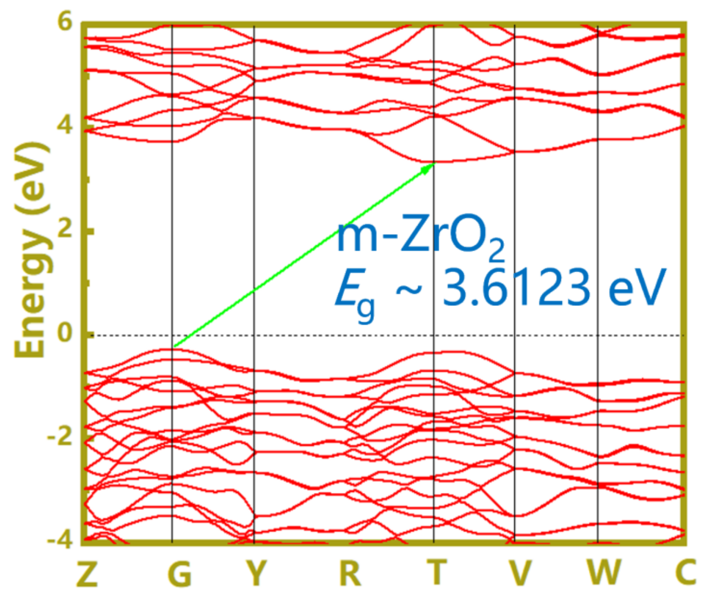

Fig. S14. Band structure diagram of Monoclinic ZrO_2.

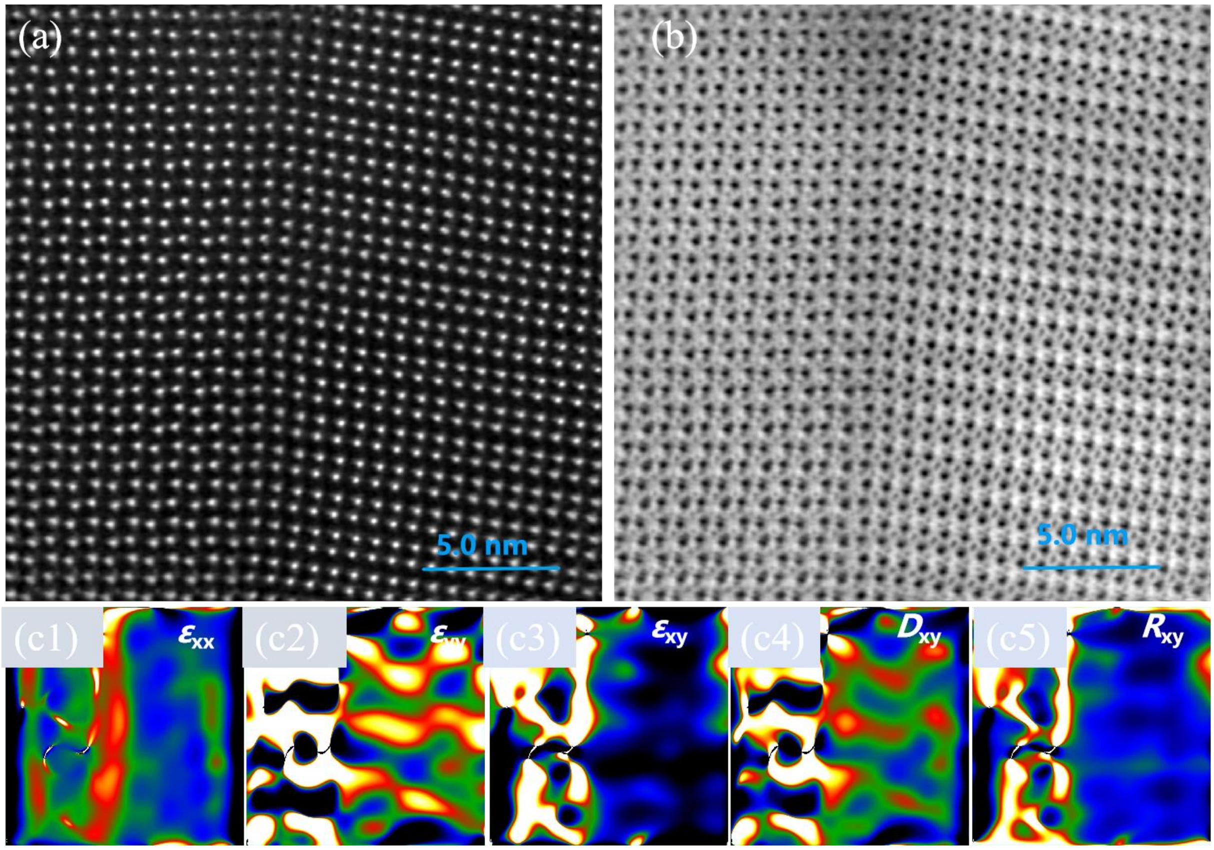

Fig. S15. (a) HADDF, (b) ABF and (c1, c2, c3, c4, c5) GPA images of ZrO_2.

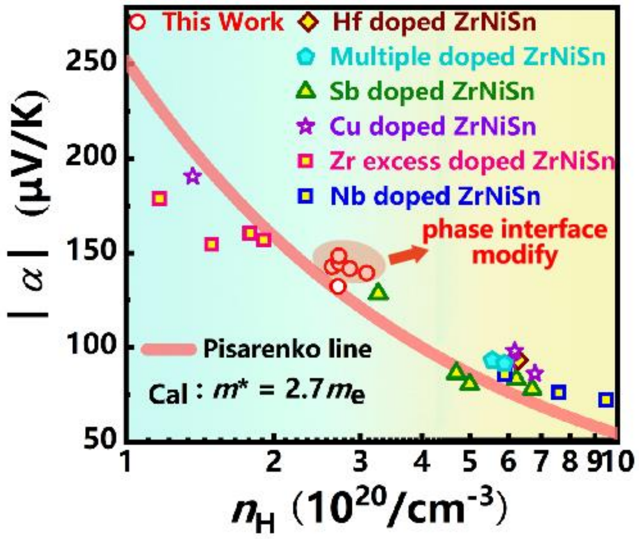

Fig. S16. Pisarenko relationship of α and component-dependent m^*

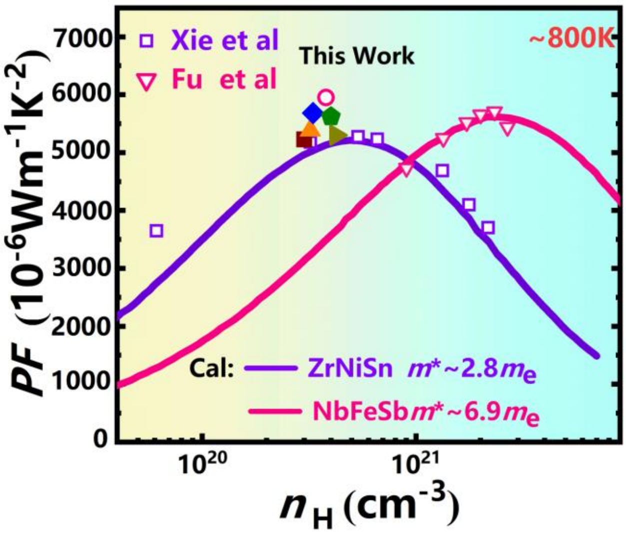

Fig. S17. Carrier concentration dependence of power factor for *n*-type ZrNiSn, *p*-type FeNbSb near 800 K.

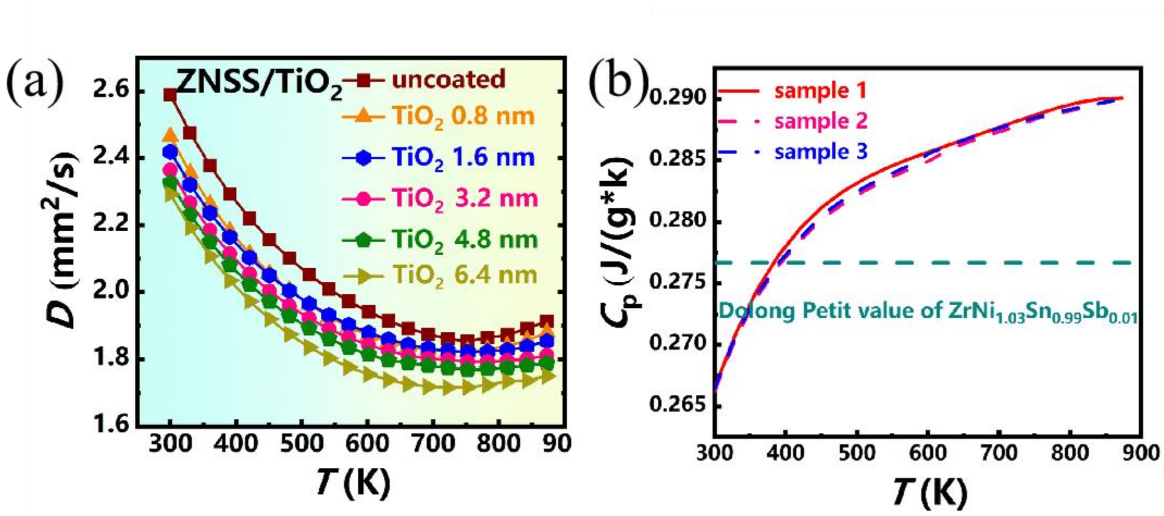

Fig. S18. (a) Temperature dependence of Thermal diffusion coefficient (D) with different ALD coated cycles. (b) Temperature dependent specific heat capacity C_p for ZNSS.

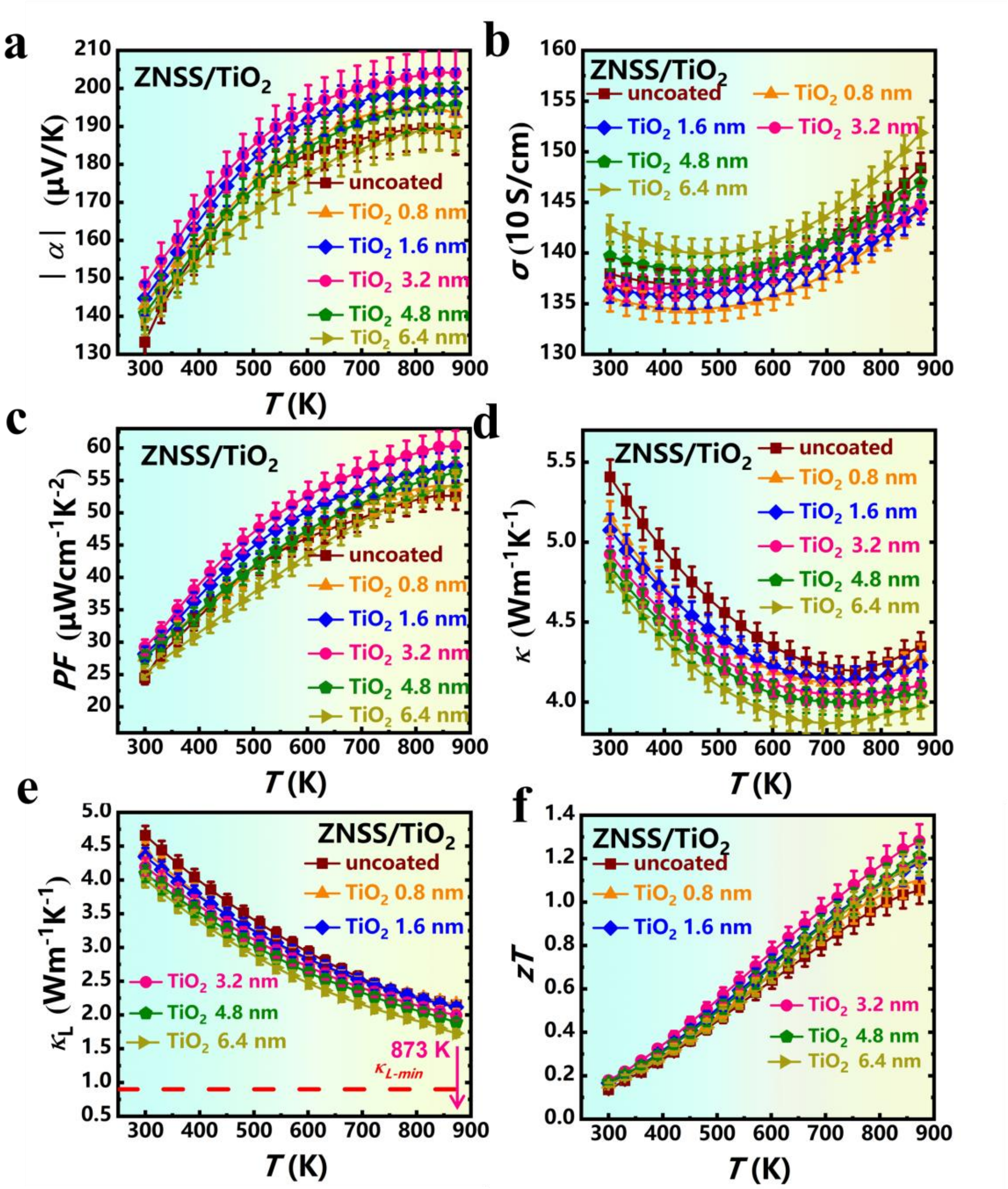

Fig. S19 Electrical and thermal transport properties with error lines. The electrical and thermal properties of the ZNSS samples before and after different ALD cycles, where uncoated, TiO_2 = 0.8 nm, TiO_2 = 1.6 nm, TiO_2 = 3.2 nm, TiO_2 = 4.8 nm, TiO_2 = 6.4 nm. (a) α, (b) σ, (c) *PF*, (d) κ, (e) κ_L and (f) *zT*. Error bars were estimated from the repeatability of the experimental result; three measurements were carried out for each material.

After applying over 40 cycles of ALD, there is a slight increase in the carrier concentration. This enhancement maybe due to the incorporation of additional Ti atoms into

the ZNSS matrix during the TiO_2 deposition process, which mildly boosts the carrier concentration.

Regarding the observed decrease in carrier mobility, while coherent interfaces theoretically reduce carrier scattering, an excessive number of such interfaces—particularly with increased ALD cycles—can act as scattering centers. This exacerbates carrier scattering and negatively impacts carrier mobility.

Despite these observations, the overall effect on electrical conductivity remains modest, primarily due to the balance between the benefits of Ti doping and the drawbacks of interface scattering.

Table Information

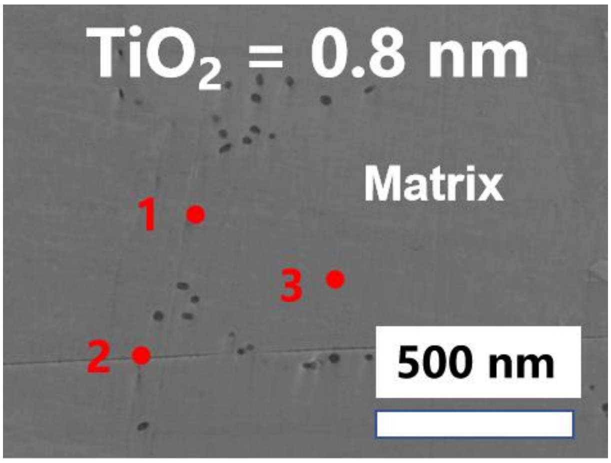

Table S1. Point-scanned data in EPMA image of TiO_2 = 0.8 nm sample (1, 2 and 3 points).

position	Zr at%	Ni at%	Sn at%	Ti at%	Sb at%
1	30.4	33.8	32.9	2.6	0.3
2	30.7	33.3	32.7	3.0	0.3
3	32.2	34.2	33.2	0.000	0.4

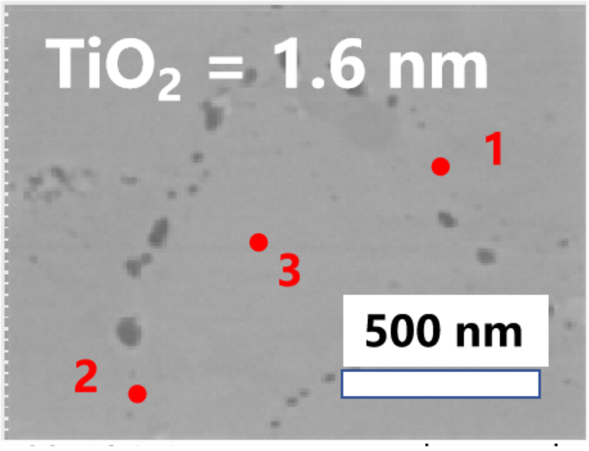

Table S2. Point-scanned data in EPMA image of TiO_2 = 1.6 nm sample (1, 2, 3 points).

position	Zr at%	Ni at%	Sn at%	Ti at%	Sb at%
1	26.7	33.7	33.1	6.2	0.3
2	26.2	33.9	33.3	6.3	0.3
3	32.2	34.2	33.2	0.0	0.4

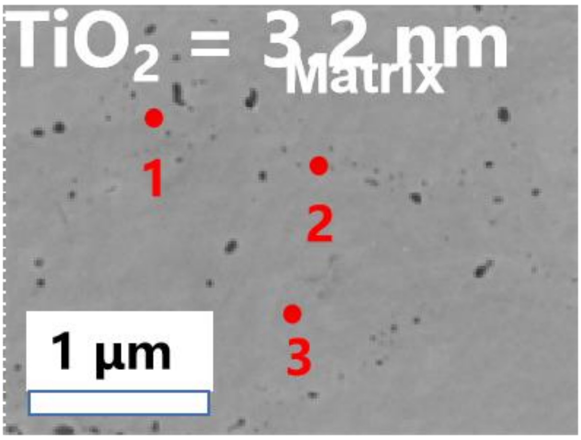

Table S3. Point-scanned data in EPMA image of TiO_2 = 3.2 nm sample (1, 2, 3 points).

position	Zr at%	Ni at%	Sn at%	Ti at%	Sb at%
1	23.8	33.1	32.9	9.9	0.3
2	23.5	33.3	33.9	10.0	0.3
3	33.1	33.6	33.0	0.0	0.3

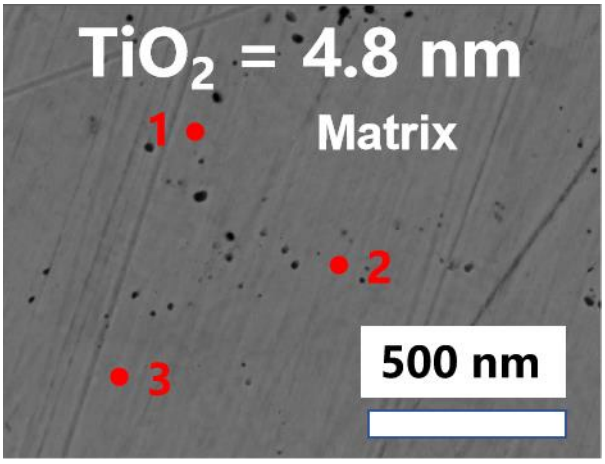

Table S4. Point-scanned data in EPMA image of TiO_2 = 4.8 nm sample (1, 2, 3 points).

position	Zr at%	Ni at%	Sn at%	Ti at%	Sb at%
1	22.6	33.8	32.1	11.3	0.2
2	22.1	33.4	32.6	11.7	0.2
3	32.7	34.1	33.0	0.0	0.2

Table S5. The normial composition and the density for uncoated, TiO_2 = 0.8 nm, TiO_2 = 1.6 nm, TiO_2 = 3.2 nm, TiO_2 = 4.8 nm, TiO_2 = 6.4 nm samples.

Norminal Composition	Density
$ZrNi_{1.03}Sn_{0.99}Sb_{0.01}$	7.836
$ZrNi_{1.03}Sn_{0.99}Sb_{0.01}$($TiO_2$ = 0.8 nm)	7.845
$ZrNi_{1.03}Sn_{0.99}Sb_{0.01}$($TiO_2$ = 1.6 nm)	7.871
$ZrNi_{1.03}Sn_{0.99}Sb_{0.01}$($TiO_2$ = 3.2 nm)	7.818
$ZrNi_{1.03}Sn_{0.99}Sb_{0.01}$($TiO_2$ = 4.8 nm)	7.817
$ZrNi_{1.03}Sn_{0.99}Sb_{0.01}$($TiO_2$ = 6.4)	7.835

Table S6. TiO_2 layer thickness of ZrNiSn/C (0, 10, 20, 40, 60, 80) samples.

Norminal Composition	TiO_2 thickness (nm)
$ZrNi_{1.03}Sn_{0.99}Sb_{0.01}$	0
$ZrNi_{1.03}Sn_{0.99}Sb_{0.01}$(C = 10)	0.8
$ZrNi_{1.03}Sn_{0.99}Sb_{0.01}$(C = 20)	1.6
$ZrNi_{1.03}Sn_{0.99}Sb_{0.01}$(C = 40)	3.2
$ZrNi_{1.03}Sn_{0.99}Sb_{0.01}$(C = 60)	4.8
$ZrNi_{1.03}Sn_{0.99}Sb_{0.01}$(C = 80)	6.4

Supplementary References

1. Wang, Y. *et al.* Role of point defects on the reactivity of reconstructed anatase titanium dioxide (001) surface. *Nat. Commun.* **4**, 2214 (2013).
2. Downie, R. A., Barczak, S., Smith, R. & Bos, J.-W. G. Compositions and thermoelectric properties of XNiSn (X= Ti, Zr, Hf) half-Heusler alloys. *J. Mater. Chem. C* **3**, 10534-10542 (2015).
3. Kresse, G. & Furthmüller, J. Efficient iterative schemes for ab initio total-energy calculations using a plane-wave basis set. *Phys. Rev. B* **54**, 11169 (1996).

4. Kresse, G. & Joubert, D. From ultrasoft pseudopotentials to the projector augmented-wave method. *Phys. Rev. B* **59**, 1758 (1999).
5. Perdew, J. P., Burke, K. & Ernzerhof, M. Generalized gradient approximation made simple. *Phys. Rev. Lett.* **77**, 3865 (1996).
6. Monkhorst, H. J. & Pack, J. D. Special points for Brillouin-zone integrations. *Phys. Rev. B* **13**, 5188 (1976).
7. Xie, H. *et al.* The intrinsic disorder related alloy scattering in ZrNiSn half-Heusler thermoelectric materials. *Sci. Rep* **4**, 6888 (2014).
